# Open revascularization for chronic mesenteric ischemia in the endovascular era: a quaternary-center experience and management algorithm

**DOI:** 10.1590/1677-5449.202301482

**Published:** 2024-02-12

**Authors:** Bruno Pagnin Schmid, Vinícius Adorno Gonçalves, Lucas Marcelo Dias Freire, Felipe Nasser, Fábio Hüsemann Menezes

**Affiliations:** 1 Universidade Estadual de Campinas - UNICAMP, Campinas, SP, Brasil.; 2 Hospital Israelita Albert Einstein - HIAE, São Paulo, SP, Brasil.

**Keywords:** mesenteric ischemia, superior mesenteric artery, mesenteric arteries, mesenteric vascular occlusion, blood vessel prosthesis, isquemia mesentérica, artéria mesentérica superior, artérias mesentéricas, oclusão vascular mesentérica, prótese vascular

## Abstract

**Background:**

Chronic mesenteric ischemia (CMI) is a debilitating disease with a heavy burden on quality of life. Stenting of the superior mesenteric artery (SMA) is the first option for treatment, but there is a lack of consensus defining precise indications for open revascularization (OR).

**Objectives:**

To describe a series of 4 patients with CMI treated with OR and to present an algorithm for the management of this condition.

**Methods:**

Three patients presented with typical intestinal angina and weight loss. One patient was subjected to prophylactic revascularization during open abdominal aortic aneurysm repair. Surgical techniques included: 1) Bypass from the infrarenal aorta to the SMA; 2) Bypass from an aorto-bifemoral polyester graft to the SMA; 3) Bypass from the right iliac artery to the SMA; 4) Bypass from the right graft limb of an aorto-biiliac polyester graft to the median colic artery at Riolan’s arcade. PTFE was used in all surgeries. All grafts were placed in a retrograde configuration, tunneled under the left renal vein, making a smooth C-loop. A treatment algorithm was constructed based on the institution’s experience and a review of recent literature.

**Results:**

All patients demonstrated resolution of symptoms and recovery of body weight. All grafts are patent after mean follow-up of two years.

**Conclusions:**

Open revascularization using the C-loop configuration is a valuable technique for CMI and may be considered in selected cases. The algorithm constructed may help decision planning in other quaternary centers.

## Resumo

**Contexto:**

A isquemia mesentérica crônica (IMC) é uma doença debilitante, com grave impacto na qualidade de vida. A literatura recomenda a angioplastia com stent da artéria mesentérica superior (AMS) como primeira opção de tratamento, mas há falta de consenso que defina indicações precisas para a revascularização aberta.

**Objetivos:**

Descrever uma série de quatro pacientes com IMC, tratados com revascularização aberta, e apresentar um algoritmo para o manejo dessa condição.

**Métodos:**

Três pacientes apresentaram angina intestinal típica e perda ponderal. Uma paciente foi submetida a reparo aberto de aneurisma da aorta abdominal e apresentava obstrução da AMS, que foi revascularizada profilaticamente. As técnicas cirúrgicas incluíram: 1) enxerto entre a aorta infrarrenal e a AMS; 2) enxerto entre o dácron utilizado em um enxerto aortobifemoral e a AMS; 3) enxerto entre a artéria ilíaca comum direita e a AMS; e 4) enxerto entre o ramo direito do dácron utilizado em um enxerto aorto-biilíaco e a artéria cólica média (ao nível da arcada de Riolan). Todos os enxertos foram feitos utilizando politetrafluoretileno em uma configuração retrógrada, tunelizados abaixo da veia renal esquerda, fazendo uma alça em C.

**Resultados:**

Todos os pacientes demonstraram resolução dos sintomas e ganho ponderal. Todos os enxertos se mantiveram pérvios durante um seguimento médio de 2 anos.

**Conclusões:**

A revascularização aberta para IMC utilizando-se a alça em C é uma técnica valiosa e pode ser considerada em pacientes selecionados. O algoritmo proposto pode auxiliar na decisão terapêutica em centros quaternários.

## INTRODUCTION

Chronic mesenteric ischemia (CMI) is a debilitating disease with a heavy burden on the quality of life of affected patients.^1^ There is also a risk of transformation into acute mesenteric ischemia, which is a catastrophic situation with a high mortality rate if not immediately treated.^1,2^

The recent literature recommends stenting of the SMA as the first line treatment because of its lower hospital morbidity and mortality and lower cost, but open revascularization may be preferred in selected cases.^3-6^

However, there is a lack of consensus defining precise indications for open revascularization in chronic mesenteric ischemia and creation of a simple algorithm to help this decision is paramount.^1,3-6^

The aim of this study is to describe a series of 4 patients with CMI who were treated with open revascularization and to present an algorithm for optimal management of this condition.

## METHODS

This study was approved by the Ethics Committee of the Institution. (CAAE 61365322.8.0000.5404, Consolidated Opinion: 5.700.372). All patients signed an informed consent form.

This is a retrospective, multicenter series of 4 consecutive cases (2 men, 2 women, mean age= 62.3 years, range 60-68 years) operated from February 2017 to February 2018 at 2 teaching hospitals in Brazil. Three patients presented with typical intestinal angina with significant weight loss in a relatively short period of time. One patient was operated for an abdominal aortic aneurysm and presented obstruction of the superior mesenteric artery and celiac trunk; she underwent prophylactic revascularization. One patient had undergone a previous unsuccessful superior mesenteric artery angioplasty. The patients’ characteristics are shown in Table 1.

**Table 1:** Patients’ characteristics.

Patient	1	2	3	4
Age (years)	60	61	68	60
Gender	Male	Male	Female	Female
Hypertension	Yes	Yes	Yes	No
Tobacco use (pack-years)	80	50	60	40
Type 2 Diabetes Mellitus	No	Yes	No	No
Dyslipidemia	No	Yes	Yes	No

The patients’ symptoms and anatomic findings are summarized in Table 2.

**Table 2:** Patients’ symptoms and anatomic findings.

Patient	1	2	3	4
Duration of symptoms (months)	1	6	6	1
Weight loss (Kg)	8	15	Yes, not measured	No
Typical angina	Yes	Yes	Yes	No
Diarrhea	Yes	No	No	No
Arterial diseases	No	PAOD, carotid endarterectomy, renal artery occlusion, CABG	TAAA	AAA, renal artery angioplasty
Mesenteric arteries	Three trunk occlusions, RA is filled by pelvic circulation	Long SMA and IMA occlusions. Poor collaterals	CT stenosis, long SMA and IMA occlusions	Long occlusions of 3 trunks, RA is filled by the LIIA

AAA = abdominal aortic aneurysm, CABG = Coronary artery bypass graft, CT = celiac trunk, IMA = inferior mesenteric artery, LIIA=left internal iliac artery, PAOD = peripheral arterial occlusive disease, RA = Riolan’s arcade, SMA = superior mesenteric artery, TAAA = thoracoabdominal aortic aneurysm.

All operations were performed through a median laparotomy with general anesthesia and the patient in supine position. All procedures were headed by an experienced vascular surgeon assisted by 3 vascular surgery residents (4 years into specialized training). All grafts were placed in a retrograde configuration, tunneled under the left renal vein and caudal to the mesocolon mesentery, making a smooth C-loop, and were anastomosed using a polypropylene continuous suture to the superior mesenteric artery in an antegrade configuration, and in one case to the median colic artery, as previously described.^7^ Systemic heparinization with 5000 IU of non-fractioned heparin was performed systematically 5 minutes before arterial clamping. Detailed surgical procedures are shown in Table 3.

**Table 3:** Details of surgical procedures.

Patient	1	2	3	4
Surgical procedure	Reinforced PTFE graft from the infrarenal aorta to the SMA	Polyester aortobifemoral graft with a reinforced PTFE graft from the graft to the SMA	Reinforced PTFE graft from the right iliac artery to the SMA	Polyester aortobiiliac graft for correction of an AAA with a reinforced PTFE graft from the right graft limb to the median colic artery at Riolan’s arcade

AAA = abdominal aortic aneurysm, PTFE = Polytetrafluoroethylene, RCC = red cell concentrate, SMA = superior mesenteric artery.

**Figure 1:**
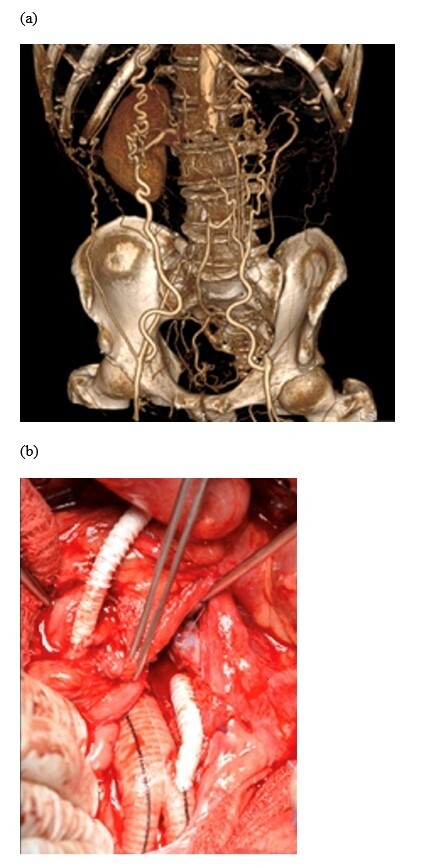
Intraoperative and imaging findings for patient #2. (a) Preoperative computed tomography angiography showing the superior mesenteric artery (SMA) and the inferior mesenteric artery occluded; (b) Intraoperative view of a polyester aortobifemoral graft with a reinforced PTFE graft from the aortic graft to the SMA.

The follow-up protocol included serial duplex ultrasound (DUS) performed by experienced radiologists at regular intervals after the procedure (at 1 month, at 1 year, and then annually). Computed tomography angiography (CTA) was performed if the US failed to assess the graft anastomosis. Figures 1 [Fig gf02] to 3 illustrate the preoperative CTA, surgical procedures, and postoperative imaging exams of some cases.

**Figure 2 gf02:**
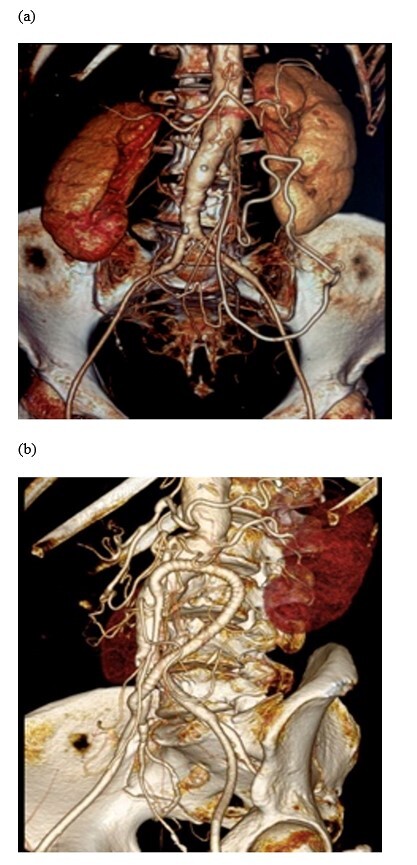
Intraoperative and imaging findings for patient #3. (a) Preoperative computed tomography angiography (CTA) with celiac trunk stenosis. The superior mesenteric artery (SMA) and the inferior mesenteric artery are occluded; (b) Postoperative CTA with a patent reinforced PTFE graft from the right iliac artery to the SMA.

**Figure 3:**
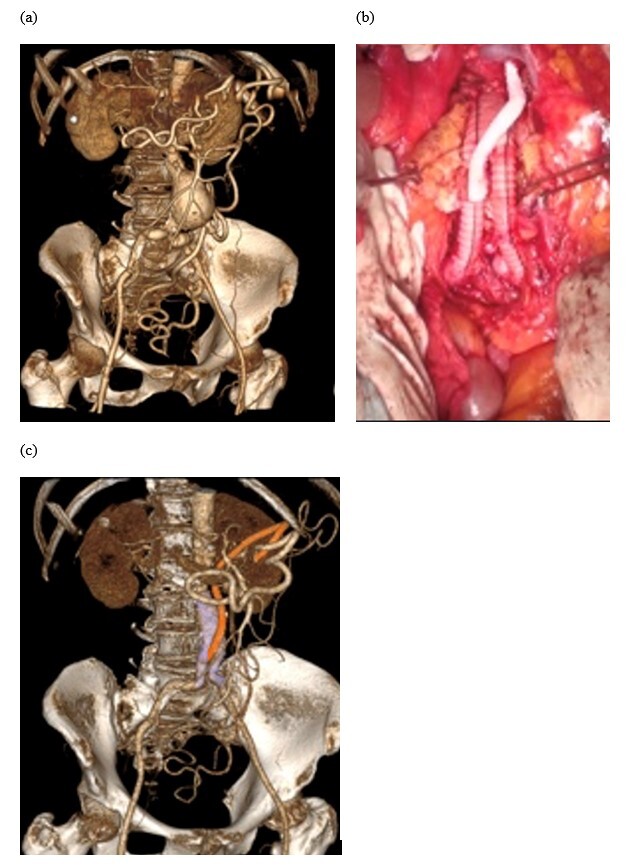
Intraoperative and imaging findings for patient #4. (a) Preoperative computed tomography angiography (CTA) with all 3 trunks occluded. Riolan’s arcade is filled by the left internal iliac artery; (b) Polyester aortobiiliac graft for correction of an abdominal aortic aneurysm with a reinforced PTFE graft from the right graft limb to the median colic artery at Riolan’s arcade; (c) Postoperative CTA showing the patent graft (highlighted in orange) and the polyester aortobiiliac graft (highlighted in purple).

## RESULTS

There were no deaths or complications in this series. All patients presented with complete resolution of the ischemic symptoms, body weight recovery, and a patent graft. Postoperatively, patients were instructed to take antiplatelet medication and statins (100mg/day of acetylsalicylic acid and 40mg/day of Simvastatin). Table 4 illustrates the patients’ outcomes.

**Table 4:** Patients’ outcomes, patency, and complications.

Patient	1	2	3	4
Re-exploration	No	No	No	No
Wound complications	No	No	No	No
Resolution of symptoms	Yes	Yes	Yes	Yes
Body weight recovery	Yes	Yes	Yes	Yes
Imaging follow-up (years)	2.91	2.24	0.08	0.8
Imaging modality	DUS	DUS	CTA	CTA
Clinical follow-up (years)	3.2	3.27	0.08	2.03
Patent graft	Yes	Yes	Yes	Yes

CTA = computed tomography angiography, DUS = duplex ultrasound.

## DISCUSSION

There is consensus in the literature to offer revascularization to patients with CMI who present symptoms of pain and weight loss.^1-3^ The typical pain described in chronic mesenteric ischemia was present in three cases in this series. Also, if there is an indication for an aortic or other abdominal procedure that could disrupt the collateral pathways, surgical reconstruction is indicated, as done in the patient in case four, who was operated prophylactically during an open AAA procedure.^1^ She presented with atypical postprandial pain, but she also gained 5kg after the operation and enlargement of the proximal segment of Riolan’s arcade was evident, as seen in the control CTA 1 year after the revascularization.

Duplex ultrasound can be used as first screening imaging modality for diagnosis of CMI.^8^ However, CTA is the primary imaging modality, with sensitivity and specificity of 95% to 100% for mesenteric arterial occlusions and it enables identification of collateral pathways.^1^ It is also indicated for evaluation of associated arterial pathologies, such as obstruction or dilatation, and is used for planning surgery.^1^ A multidisciplinary team plays a key role in the preoperative scenario, to better assess the patient’s clinical conditions and nutritional status and to confirm the diagnosis using functional tests in selected cases.^8^ At our centers, all patients were evaluated by experienced gastroenterologists, cardiologists, pneumologists, vascular surgeons, and interventional radiologists.

The endovascular-first recommendation is consistent with several practice guidelines and there are studies supporting use of routine mesenteric stenting instead of plain balloon angioplasty, specifically, use of covered stents.^9,10^

However, there is a lack of consensus regarding the exact threshold for deciding to opt for open revascularization and the greatest challenge is to select patients who will benefit from this treatment.^8,11^

Clinical risk stratification is the first step to guide the decision on treatment approach. Definitions of “high-risk” and “low-risk” patients are largely variable between studies and reporting standards are inconsistent. Use of a previously validated tool, such as the Society for Vascular Surgery score, can be helpful to establish this classification.^12^ This is a simple algorithm based on patients’ age, pulmonary and cardiac dysfunction, and renal insufficiency, in which patients are classified into low-risk or high-risk categories by the presence of at least one of the high-risk criteria defined.^12^ All of the patients in our study were classified as “low-risk” and so open revascularization could be attempted.

The second imperative principle for open revascularization is the anatomic pattern. There are some specific conditions in which the endovascular technique may be unfeasible, such as flush aortic occlusions, long segment occlusions (>2cm), severe calcification, tandem lesions, distal lesions, and small diameter vessels.^8,9^ An additional potential contraindication to the endovascular approach is the situation in which open revascularization is performed prophylactically during open procedures that could disrupt collateral pathways, such as open AAA repair with previously diagnosed mesenteric stenosis, as presented in case number four.

The endovascular technique is rapidly evolving and some authors have described endovascular strategies to overcome these barriers.^13^ Sharafuddin et al.^13^ reported their experience with 27 patients with total mesenteric vessel occlusion with promising results regarding technical success rate (85%). Nevertheless, the 1 and 3-year primary patency rates were disappointing (58% and 33% respectively). Additionally, 2 patients died in this series. On the other hand, open repair is definitely durable with a 5-year primary patency of 88% in some series.^12^ These results reaffirm that the anatomical threshold has yet to be crossed and the role of endovascular treatment for these higher risk anatomic lesions is not firmly established.^14,15^ Also, the need for reinterventions must be discussed during patient counseling in order to assist the treatment choice.^12,16,17^

The most challenging situation is to select optimal treatment in high-risk patients with high-risk anatomic lesions. Some authors support initial stenting as a bridge therapy so that open surgery can be performed later, or repeated interventions to provide short-term symptom relief for patients with short life expectancy.^17,18^

Other groups believe that the open surgical approach performed retrograde (using the iliac artery as the inflow source) and avoiding aortic clamping can be performed safely as a good option for this group of patients.^12,16^ This technical solution was performed in patient #3 with good results.

There is also a discussion regarding the number of arteries that need to be treated. When performing angioplasty, there is a tendency to treat all three trunks, even though there is a preference for the SMA.^19^ During open revascularization, the literature shows good results with revascularization solely of the SMA, with long term results similar to performing grafts for more than one trunk.^20,21^

**Figure 4:**
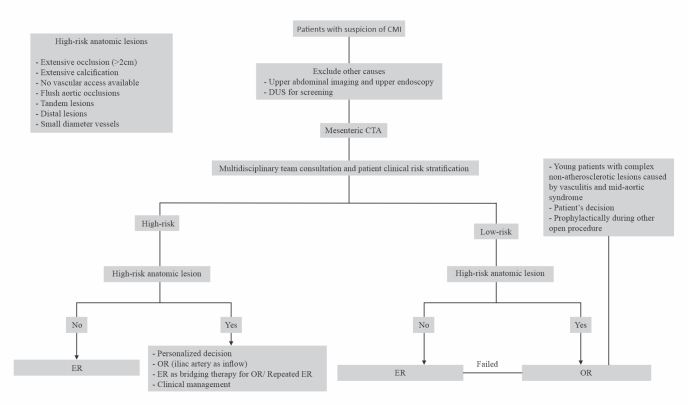
Proposed algorithm for chronic mesenteric ischemia (CMI) management. CTA = computed tomography angiography; DUS = duplex ultrasound; ER = endovascular repair; OR = open repair.

Finally, the choice of treatment approach may be not so elementary as a simple issue of endovascular or open first. The decision must involve patient clinical risk stratification by a multidisciplinary team, detailed anatomic profile, individual goals of care, surgeon experience, and patient decision. An algorithm for CMI management based on the authors’ experience and an extensive literature review is proposed in Figure 4.

In this series, all patients demonstrated resolution of the ischemic symptoms and body weight recovery. All grafts are patent after a mean follow-up of 2 years. The series draws attention to the simultaneous occurrence of other arterial manifestations related to the degenerative process of atherosclerosis, such as: renal artery, carotid, and lower limb occlusions, and the presence of dilatation of aortic segments, which is in consonance with the existing literature.^22^ Nonetheless, the open revascularization procedures achieved good results, with low procedural mortality, just as the literature indicates for selected patients, reaffirming the utmost importance of open revascularization in CMI, even in the endovascular era.^23^

The limitations of the present study include its retrospective design with a small number of patients and a relatively short follow-up period.

## CONCLUSION

Open repair using the C-loop configuration is a feasible technique for CMI with good results in selected cases. The algorithm constructed may help decision planning at other centers.
